# Attentional impairment and altered brain activity in healthcare workers after mild COVID-19

**DOI:** 10.1007/s11682-024-00851-4

**Published:** 2024-02-01

**Authors:** Keyi Lin, Yaotian Gao, Wei Ji, Yan Li, Wei Wang, Mengcheng Du, Jia Liu, Zhengyu Hong, Tao Jiang, Yuyang Wang

**Affiliations:** 1 Department of Neurosurgery, The First Affiliated Hospital of Anhui Medical University, Hefei, China; 2 Anhui Public Health Clinical Center, Hefei, China; 3 Department of Neurosurgery, Hefei Huaan Brain Hospital, Hefei, China; 4 Department of Radiology, The First Affiliated Hospital of Anhui Medical University, Hefei, China; 5 Department of Neurosurgery, The Second Affiliated Hospital of Anhui Medical University, Hefei, China; 6 Anhui Provincial Institute of Translational Medicine, Hefei, China

**Keywords:** COVID-19, Healthcare workers, Cognitive impairment, Attentional network, fMRI

## Abstract

Severe acute respiratory syndrome coronavirus 2 (SARS-COV-2) is highly transmissible and pathogenic. Patients with mild cases account for the majority of those infected with coronavirus disease 2019 (COVID-19). Although there is evidence that many patients with COVID-19 have varying degrees of attentional impairment, little is known about how SARS-COV-2 affects attentional function. This study included a high-risk healthcare population divided into groups of healthcare workers (HCWs) with mild COVID-19 (patient group, *n* = 45) and matched healthy HCWs controls (HC group, *n* = 42), who completed general neuropsychological background tests and Attention Network Test (ANT), and underwent resting-state functional magnetic resonance imaging (rs-fMRI) using amplitude of low-frequency fluctuation (ALFF) to assess altered brain activity; Selective impairment occurred in orienting and executive control networks, but not in alert network, in the patient group, and widespread cognitive impairment encompassing general attention, memory, and executive dysfunction. Moreover, the patient group had significantly lower ALFF values in the left superior and left middle frontal gyri than the HC group. SARS-COV-2 infection may have led to reduced brain activity in the left superior and left middle frontal gyri, thus impairing attentional orienting and executive control networks, which may explain the development of attentional deficits after COVID-19.

## Introduction

Severe acute respiratory syndrome coronavirus 2 (SARS-CoV-2), the causative agent of coronavirus disease 2019 (COVID-19), is highly transmissible, pathogenic, and continues to spread worldwide (Chen et al., 2022). Approximately 81% of COVID-19 cases are mild (Wu & McGoogan, 2020), and clinical attention to mild patients is mostly lacking because they are thought to recover spontaneously 1-2 weeks after COVID-19 and do not even require hospitalization. Non-hospitalized patients may face ongoing emotional, behavioral, and cognitive impairments despite the mild clinical symptoms. Current research has focused on exploring the development of cognitive and psychiatric impairments in ambulatory patients with COVID-19 (Graham et al., 2021; Schild et al., 2023). Few studies have conducted a deeper exploration and comprehensive multidimensional assessment of cognitive impairment in patients with mild COVID-19.

Cognitive impairment is a characteristic of “long-term COVID-19” syndrome (Graham et al., 2021), especially involving attention, memory, and executive function impairments. Many patients recovering from COVID-19 complain of poor concentration and memory loss. As a core component of cognitive and behavioral processes, attentional function plays a key role in basic and higher functions and has a large impact on daily life and work. The decline in attentional function may be key to cognitive impairment (Bertuccelli et al., 2022; Calabria et al., 2022; Crivelli et al., 2022; Kirchberger et al., 2023; Michelen et al., 2021). Therefore, an adequate understanding of attentional deficits in patients with a mild post-COVID-19 condition is key to improving their prognosis and quality of life.

Traditional paper-and-pencil tests, including the Trail Making Test, Stroop, WAIS Digit Span, and Continuous Performance Test, are commonly used in current research and clinical practice to assess attentional function (Tavares-Júnior et al., 2022; Zhou et al., 2020); however, they lack sensitivity and specificity. The tests can only clarify the occurrence of attentional dysfunction in patients but not accurately assess the mechanisms underlying attentional impairment in terms of attentional networks. In contrast, the standardized Attention Network Test (ANT), a computer-based test designed by Fan et al, can quickly and effectively assess functional changes in three separate attentional networks (alerting, orienting, and executive control networks) (Fan et al., 2002). The ANT has been widely used in population studies of attention deficit hyperactivity disorder, Parkinson’s disease, schizophrenia, Alzheimer’s disease, and brain injury (Arora et al., 2020; Pultsina et al., 2022; Wang et al., 2023; Yang et al., 2022).

The resting-state functional magnetic resonance imaging (rs-fMRI) is a non-invasive neuroimaging technique that reveals the intrinsic spontaneous activity of the brain through changes in magnetic resonance signals generated by altered blood oxygen levels (Zhou et al., 2010). It is increasingly used to study the neural mechanisms of various neurological disorders. Of various metrics established to study rs-fMRI data, the amplitude of low frequency fluctuations (ALFF) is one of the most commonly used, to detect local abnormal activity in specific brain regions. ALFF reflects the intensity of spontaneous synchronized neural activity of various low frequency range voxels (0.01–0.1 HZ), from the perspective of energy metabolism (Wang et al., 2020). Increased ALFF values indicate increased excitability in the brain regions. However, no study has used the ALFF index to explore changes in brain activity in patients with mild attentional deficits after COVID-19.

As a high-risk group, healthcare workers (HCWs) are continuously exposed to SARS-COV-2 infection and its consequences during clinical work. Faced with the complex social environment and high workload of pandemic prevention, we believe that even in HCWs with mild SARS-COV-2 infection, impairment of attentional function persists and affects later clinical work. Therefore, we recruited HCWs with mild SARS-COV-2 infection to explore in-depth, attentional network impairments, using ANT, to understand the changes in patients’ levels of attentional function impairments comprehensively. We also determined the changes in brain activities, to understand attentional network impairment mechanisms by combining rs-fMRI data. This finding has important implications for the early identification of attentional impairments to guide rapid rehabilitation and cognitive interventions later in life.

## Materials and methods

### Participants

The study population in this cross-sectional study included HCWs who were at high risk of SARS-COV-2 infection and had contracted COVID-19 at our center (hereafter, patient group). The inclusion criteria were: (i) patients who contracted the SARS-COV-2 from December 2022 to January 2023 as evidenced by a positive antigen staining or reverse transcription-polymerase chain reaction tests results of nasal and pharyngeal swabs, with mild symptoms in the acute phase that did not require hospitalization; (ii) no history of other influenza illnesses during the study period; (iii) age between 18 and 65 years; and (iv) no previous history of neurological or psychiatric disorder, traumatic brain injury, or brain surgery. The exclusion criteria were: (i) history of drug abuse; (ii) alcohol abuse; and (iii) serious systemic diseases such as cardiovascular, lung, and kidney diseases.

Healthy controls (HC) were HCWs recruited at the same time, were matched for age and years of education with the study group but were not infected with SARS-COV-2.

All the participants provided written informed consent. The study was conducted in accordance with the Declaration of Helsinki and approved by the local ethics committee.

### Neuropsychological background tests

The Hamilton Anxiety Rating Scale (HAMA) and Hamilton Depression Scale (HAMD) were used to assess anxiety and depressive mood, respectively. The Montreal Cognitive Assessment Test (MoCA) was used to assess general cognitive function (Huang et al., 2021). General attention was tested using the Digit Span Test (forward and backward), Stroop Color Test (Perianez et al., 2021), and Trail Making Test A. The Stroop Word Test, Stroop Interference Test, and Trail Making Test B were used to assess information processing and executive functioning. The Auditory Verbal Learning Test (AVLT) was used to assess memory (Can et al., 2016).

### Attention network test

**Fig. 1:**
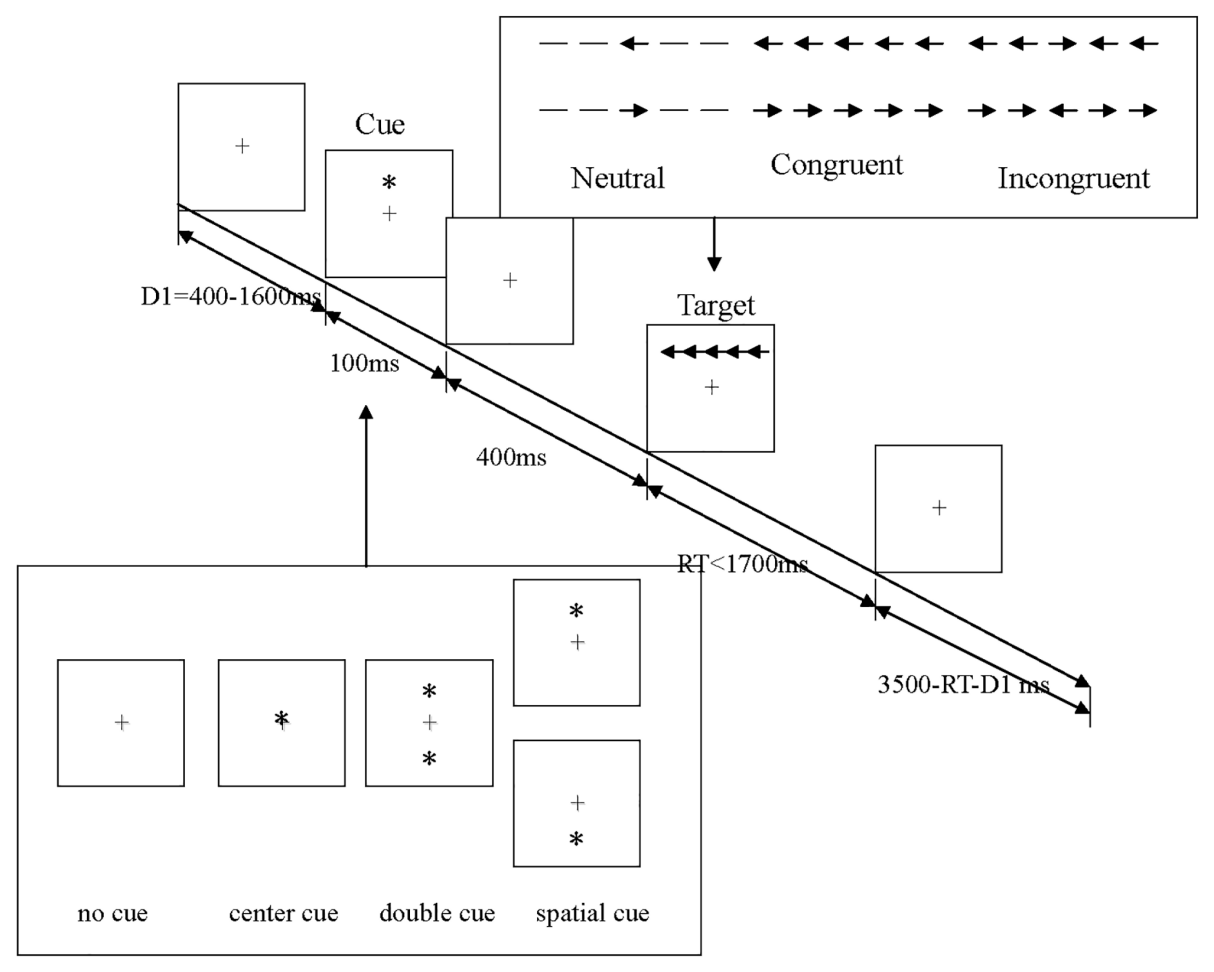
Schematic of the attention network test (ANT). (**a**) The four cue conditions. (**b**) The three flanking conditions. (**c**) An example of the procedure

The ANT is a combination of a flanking and spatial cueing task, which depends critically on the measurement of performance (reaction time [RT] and accuracy) of stimuli presented during different conditions and the calculation of different scores for alerting, orienting, and executive control (usually based on RTs) (de Souza Almeida et al., 2021). Before proceeding with the attention network test, the researchers informed the participants about the procedure, purpose, requirements, and precautions of the test. During the test, participants were placed in a quiet, separate room and asked to keep their eyes on the “+” in the center of a computer screen and their fingers on the response keys. The test consisted of a 2-min training set (to familiarize participants with the test) and three 5-min test sets, with a 3-5 min break between each set, over approximately 30 min duration. Each test set consisted of 312 trials, and each included a random “*”-like cue above, below, to the left and right of the “+” in the center of the computer screen before the target arrow appeared over an equal number of times. The cue also appeared randomly with different flanking conditions (Fig. 1). The participants were required to press the corresponding directional key quickly after the appearance of the target arrow to record the RT and accuracy rate.

### Attention network efficiency

The following parameters are calculated using the RT:

Alertness network efficiency = RT_no cue_ - RT_double cue_.

Orienting network efficiency = RT_central cue_ - RT_spatial cue_.

Execution control network efficiency = RT_incongruent_ - RT_congruent_.

Higher alerting and orienting network efficiencies corresponded to higher alerting and orienting capabilities, respectively, while higher executive control efficiency corresponded to lower executive control capabilities. The accuracy was defined as the number of correct trials divided by the total number of trials (*n* = 312). Network ratios were obtained by dividing the network efficiencies by the individual mean RT. This ratio was used to examine specific effects that were not affected by the overall RT differences.

### Functional magnetic resonance imaging data acquisition

All MRI data were collected using a 3.0T Philips MRI scanner (Discovery Achieva; Philips Healthcare, Holland) with an 8-channel magnetic head coil. The participants were required to remain supine with their bodies still, eyes closed, and not think about anything in particular, throughout the scanning duration. In addition, they were also required to wear earplugs and place a foam pad between their head and the coil, to minimize motion artifacts caused by head movements. High-resolution 3D T1-weighted structural images were acquired using turbo field-echo (TFE) sequence with repetition time (TR) = 8.2 ms; echo time (TE) = 3.8 ms; field of view (FOV) = 256 mm × 256 mm; matrix size = 256 × 227; slice thickness = 1 mm, no gaps; 188 sagittal slices; acquisition time = 332 s. Resting state BOLD fMRI data were acquired using echo planar imaging (EPI) sequence including the following parameters: TR = 2000 ms; TE = 30 ms; FOV = 220 mm × 220 mm; matrix size = 64 × 65; slice thickness = 3 mm, slice gap = 1 mm; 35 interlaced axial slices; and acquisition time = 399 s.

### Functional magnetic resonance imaging data preprocessing

Functional images were preprocessed using the fMRI Data Processing Assistant (http://rfmri.org/DPARSF) (Chao-Gan & Yu-Feng, 2010), WhiteMatter (Ji et al., 2019), and SPM12 (http://www.fil.ion.ucl.ac.uk/spm/software/spm12). The first five functional volumes were removed, sliced, and the remaining images were rearranged. The structural images were then co-aligned with these preprocessed functional images and segmented into gray matter (GM), white matter (WM), and cerebrospinal fluid (CSF) using differential anatomical alignment with Exponentiated Lie Algebra (DARTEL) (Ashburner, 2007). Based on the transformation matrix generated using DARTEL, the CSF mask in the Montreal Neurological Institute (MNI) space (70% threshold on the SPM12 prior probability map) was transformed into individual functional spaces. The mean signal and 24 head motion parameters (Friston et al., 1996) in CSF mask were regressed from the functional images in each participant individual space. Individual masks were generated on the probability maps of the GM and WM (generated by structural segmentation) using thresholds of 50% and 90%, respectively. The functional images were spatially separated into GM and WM images based on these two masks. The GM image was normalized to MNI space by structural segmentation and smoothed (4 mm full-width half-maximum, isotropic) and filtered (0.01 ~ 0.1 Hz). ALFF was computed and normalized by zero-mean normalization.

### Statistical analyses

Statistical data were analyzed using IBM SPSS Statistics for Windows, version 26.0 software (IBM Corp., Armonk, NY, USA). Our data followed a normal distribution, were analyzed using independent samples t-tests, and evaluated to identify differences between patients and matched HCs. Pearson’s correlation coefficients and false discovery rate (FDR) corrections were used to assess the relationship between network efficiency, neuropsychological background test scores and ALFF values. The significance level for all tests was set at *P* < 0.05.

Between-group comparisons of imaging data were performed for alignment using the SPM12 toolbox with statistical nonparametric mapping (SnPM). Age, sex, and educational level were used as covariates. Briefly, each trial was randomly assigned a label (“patient” or “control”) and repeated 5000 times. For each trial, a two-sample t-test was used to generate a t-plot. Based on the distribution of these 5000 t-plots, it was possible to infer whether the t-values in the true labeling condition were significant (Nichols & Holmes, 2002). To control for errors in multiple comparisons, we first set a cluster-defined threshold of *P* = 0.001. Only clusters larger than a given capacity were reported as having survived the clusters-level correction (Pcorr < 0.05).

## Results

### Neuropsychological background tests

Fifty HCWs with mild COVID-19 and 42 HCs were included in this study. Of the 50, 5 HCWs were excluded due to emotional disturbance and inability to cooperate. The demographic characteristics and neuropsychological test results of the included participants are shown in Table 1. Differences in age, educational level, MoCA, HAMA, and HAMD scores between the patients and HC groups were not statistically significant (*P* > 0.05). In the tests of attention and memory, information processing and executive function, for the patient group, Trail Making A and B, immediate and delayed recall, and the Stroop Word Test were also not statistically significant (*P* > 0.05). However, the WAIS Digit Span Test (forward and backward), Stroop Color Test, Recognition and Stroop Inference Test in patient group were statistically different from those of the HC group (*P* < 0.05). Summarily, the patient group had cognitive impairments in executive function, memory recognition, and attention based on neuropsychological background tests.

**Table 1:** Demographic characteristics and summary of neuropsychological test of patients and healthy controls

	Patient group (*n* = 45)	HC group (*n* = 42)	t	*P*
	Mean or Count (SD)	Mean or Count (SD)		
Age(years)	31.31(7.504)	32.43(10.421)	-0.570	0.570
Education(years)	15.60(3.172)	14.38(2.686)	1.972	0.057
HAMD	3.20(3.727)	3.00(2.00)	0.315	0.754
HAMA	2.38(3.359)	2.31(1.774)	0.120	0.905
MoCA	28.60(1.355)	27.98(2.124)	1.644	0.104
Attention/concentration				
WAIS Digit Span(forward)	7.00(1.261)	7.95(0.216)	-4.988	<0.01^b^
WAIS Digit Span(backward)	5.84(1.127)	6.69(0.517)	-4.548	<0.01^b^
Stroop Color Test (sec)	18.22(4.557)	15.91(3.675)	2.598	0.011^a^
Trail Making A (sec)	37.448(10.767)	41.163(9.065)	-1.734	0.086
Memory (AVLT)				
Immediate Recall	12.33(2.403)	12.14(2.055)	0.396	0.693
Delayed Recall	12.40(2.368)	11.43(2.441)	1.884	0.063
Recognition	11.00(3.020)	12.64(2.197)	2.887	<0.01^b^
Information Processing and Executive function				
Trail Making B (sec)	86.59(22.325)	92.440(19.337)	-1.302	0.196
Stroop Word Test (sec)	19.82(5.167)	18.52(3.258)	1.394	0.167
Stroop Interference Test (sec)	34.69(10.204)	30.17(6.848)	2.408	0.018^a^

Abbreviations: HC, healthy control; SD, standard deviation; HAMA, Hamilton Anxiety Rating Scale; HAMD, Hamilton Depression Rating Scale; MoCA, Montreal Cognitive Assessment Test; WAIS, Wechsler Adult Intelligence Scale; AVLT, Auditory Verbal Learning Test. Sec, seconds

^a^ compared to HC group (*p* < 0.05); ^b^ compared to HC group (*p* < 0.01)

### Attention network efficiency

**Fig. 2:**
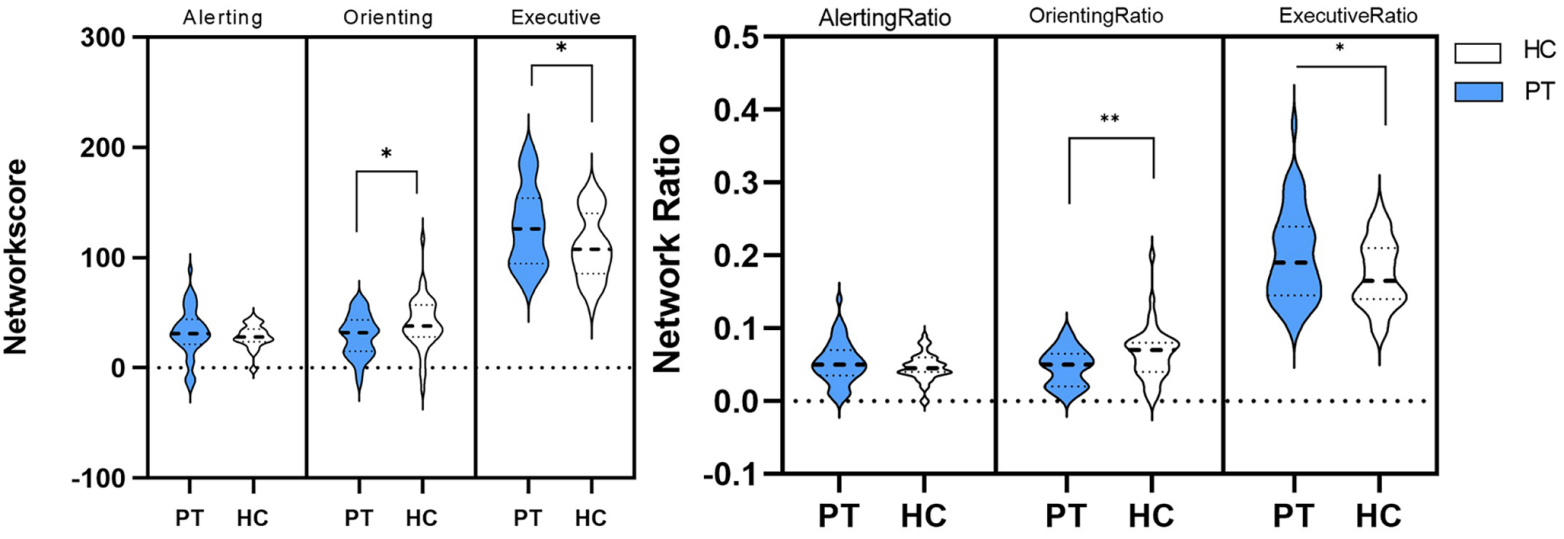
Network scores and network ratio scores for patient and healthy controls. Blue represents the patient group and white represents the HC group, where the thicker dotted line represent the median and the thinner dotted line represent the quartile. [* indicates *p* < 0.05, ** indicates *p* < 0.001]

Table 2 shows no significant difference between patients and HC groups in alert network efficiency but shows significantly lower orienting network efficiency and significantly higher executive control network efficiency in patient group than that in HC group (*P* < 0.05); that is, patient group had reduced attentional orienting and executive control abilities. The network and network ratio scores of the patient and HC groups are shown in Fig. 2. The patient group had significantly lower orienting network ratios and significantly higher executive control network ratios than the control group (Table 2). In addition, differences in mean RT and accuracy between the patient and HC groups were not statistically significant (*P* > 0.05).

**Table 2:** Attention performance of patients and healthy controls

Performance	Patient group (*n* = 45)	HC group (*n* = 42)	t	*P*
	Mean or Count (SD)	Mean or Count (SD)		
Alerting	32.40(22.113)	28.36(10.596)	1.099	0.276
Ratio	0.05(0.03)	0.05(0.02)	0.771	0.479
Orienting	29.18(18.723)	40.21(23.847)	-2.410	0.018^a^
Ratio	0.04(0.03)	0.07(0.04)	-2.850	<0.01^b^
Executive	128.16(36.113)	110.17(30.377)	2.505	0.014^a^
Ratio	0.19(0.06)	0.17(0.05)	2.104	0.038^a^
Mean RT	650.76(68.743)	633.62(73.098)	1.127	0.263
Accuracy (%)	97.24(3.325)	98.26(1.547)	-1.809	0.074

The scores of three groups in the table are the derived scores. HC, healthy control; RT, reaction time; SD, standard deviation

^a^ compared to HC group (*p* < 0.05); ^b^ compared to HC group (*p* < 0.01)

### Functional magnetic resonance imaging results

**Fig. 3:**
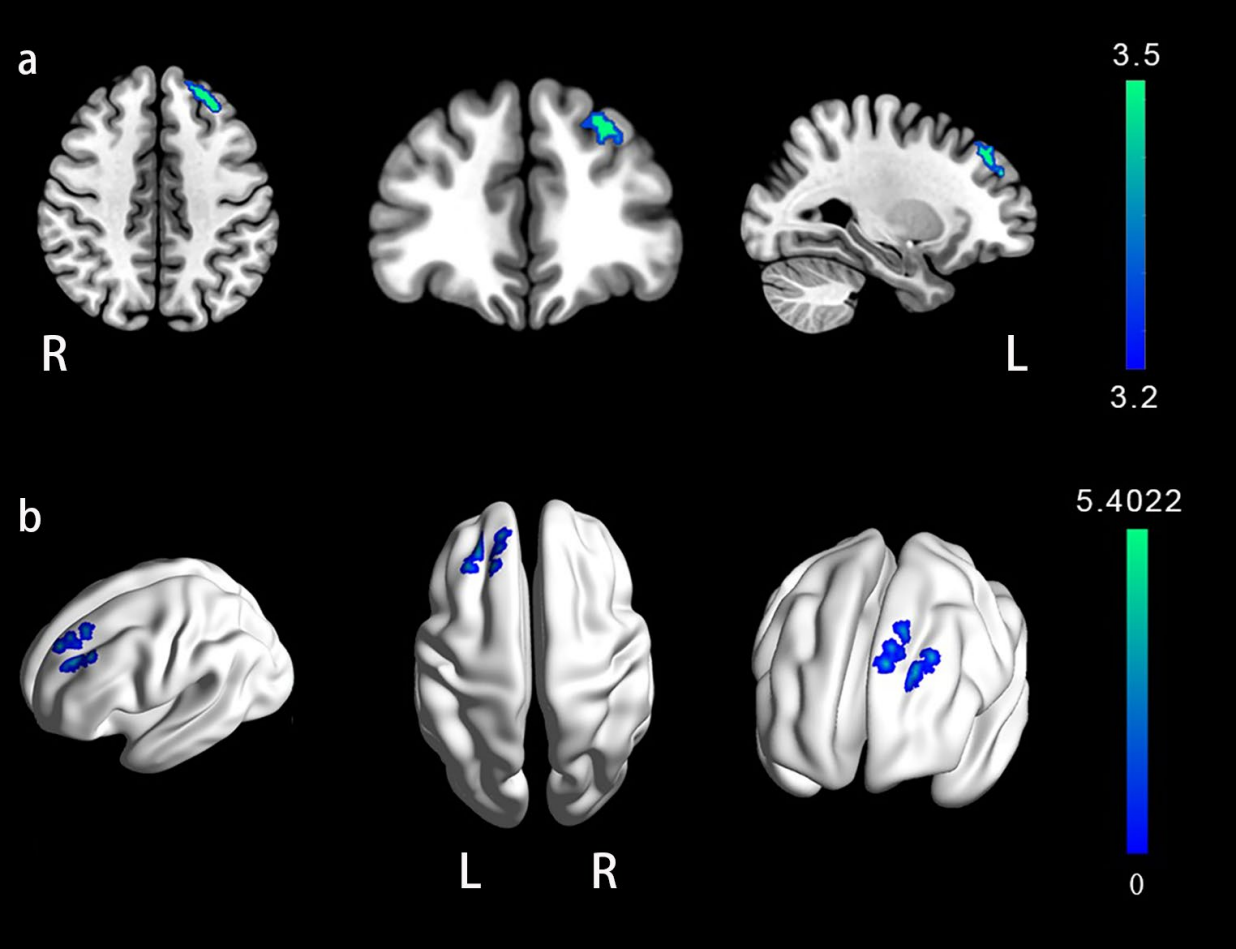
Statistical results of rs-fMRI. The brain regions with reduced ALFF values in mild COVID-19 patients compared with HC group were mainly located in the left superior and the left middle frontal gyri (blue-green areas in Fig. 3). **a**. The MRIcronGL software was used to overlay the results of the ALFF indexed differential brain regions using the standard mni152 template as a base plate. The peaks in the cluster (peak coordinates: -30, 36, 42, peak t-value: 5.4022). **b**. BrainNet software was used to overlay the results of brain regions showing reduced ALFF values, using the ICBM152 template as a base plate. The color bar represents t-values. R, right; L, Left

In this study, the ALFF was used to describe the intensity of local brain activity. We found significantly lower ALFF values in the patient group in the left superior and left middle frontal gyri compared to that in the HC group (Fig. 3). The left superior frontal gyrus peaked in this cluster (Peak coordinate: -30,36,42; Peak t-value: 5.4022).

### Correlation analysis

In the patient group, we observed no significant correlation between attention network efficiency, neuropsychological background test results or ALFF values (*P* > 0.05).

## Discussion

In this study, we investigated the differences in cognitive function between the patient and HC group and assessed the alterations in three separable attentional networks (i.e., alerting network, orienting network, and executive control network) using the ANT, as well as the altered brain activities in the patient group using rs-fMRI based on the ALFF metric. The main findings were as follows: (i) compared with HC group, the patient group showed significant differences in WAIS Digit Span Test (forward and backward), Stroop Color Test, Recognition and Stroop Inference Test. These indicate impairments in general attention, memory, and executive function in the patient group. (ii) Alerting network efficiency was not significantly different between the patient group and the healthy control group, but orienting network efficiency was significantly lower and executive control network efficiency was significantly higher in the patient group compared to the control group. That is, the patient group had decreased attentional orienting and executive control. (iii) Brain activities in the left superior and left middle frontal gyri were significantly lower in the patient group than in the HC group.

Our study found varying degrees of reduced efficiency in both the attentional orienting and executive control networks in the patient group. The corresponding neuropsychological background test results also indicated the presence of impairments in general attention and executive function. However, the present study did not find a strong correlation between the decrease in general attentional and executive functions and the efficiency of the two attentional subnetworks. We believe that the current study population, including HC group, were highly educated and highly intelligent individuals whose decline in general cognitive function was relatively subtle (Steward et al., 2018) and may require more specific neuropsychological methods for assessment in the future.

The disruption in orienting and executive control networks may manifest through several closely related mechanisms. First, the impairment of frontal lobe function and abnormal functional connectivity between networks in patients with COVID-19, together with the extensive composition of the brain regions in orienting and executive control networks, gives us reason to believe that orienting and executive control networks were also affected. The orienting network depends on the joint regulation of the dorsal and ventral attention networks and is mainly distributed in the bilateral intraparietal sulcus, frontal middle gyrus, frontal eye field, and middle temporal gyrus (Farrant & Uddin, 2015). In contrast, the executive control network relies on the anterior cingulate cortex, prefrontal cortex and subcortical structures such as the basal ganglia and cerebellum as well as their projections into the entire cortex (Markett et al., 2022; Sarrias-Arrabal et al., 2023), mainly in the anterior cingulate gyrus, middle frontal gyrus, superior frontal gyrus, and thalamus (Matsumoto & Tanaka, 2004). A longitudinal voxel-based ^18^F Fluorodeoxyglucose positron emission tomography (18 F-FDG-PET) study demonstrated persistent hypometabolism of prefrontal lobes in COVID-19 patients with attentional disorders (Kas et al., 2021). Similarly, our rs-fMRI results showed significantly lower ALFF values in the left superior and left middle frontal gyri in the patient group than in the HC group. As an overlapping brain region of the orienting and executive control networks, a decrease in the frontal cortex activity directly affects attentional network efficiency. At the same time, the study confirmed abnormal alterations in functional connectivity between the salient network, dorsal attentional network, and default mode network in patients with attentional deficits after COVID-19 (Paolini et al., 2023). A study of functional brain networks in patients with mild COVID-19 also found that non-hospitalized COVID-19 patients had significantly and uniformly reduced functional connectivity within and between the temporal lobe and subcortical regions, including the thalamus, parahippocampal gyrus, amygdala, basal ganglia, and superior temporal gyrus (Churchill et al., 2023). Of these, the thalamus and basal ganglia have been shown to be involved not only in executive control but also in the regulation of attentional orientations (Xuan et al., 2016). Therefore, we speculated that altered functional connectivity within or between functional brain networks may contribute to attentional impairment. Recently, an increasing number of studies have identified interactions between attentional subnetworks that may contribute to the synergistic reduction of orienting and executive control networks (Xuan et al., 2016). In particular, studies have confirmed that attentional orienting network can increase the efficiency of the executive control network to support attentional functioning (Callejas et al., 2004).

There was no extensive damage to the brain microstructure in these patients with mild SARS-COV-2 infection. One study showed no abnormalities in head MRI scans and angiograms in mild COVID-19 patients (Ohtake et al., 2023). ANT results showed only attentional orienting and executive control network impairment and no changes in alerting networks in HCWs with mild COVID-19. Studies have confirmed that patients with mild COVID-19 tend to show only a reduction in the volume and length of WM fiber tracts compared to patients with severe COVID-19 who show a reduction in cortical thickness, cerebral blood flow, and WM fiber tracts (Qin et al., 2021). Studies have also found only a small proportion of frontal cortical atrophy and periventricular WM hyperintensities in non-hospitalized patients (Bungenberg et al., 2022). Therefore, we speculate that milder damage to the brain microstructure in HCWs with mild COVID-19 did not cause extensive damage to the attention network. However, we did not identify a correlation between SARS-COV-2 damage to the brain microstructure and attentional network alterations, and further studies are needed to confirm this finding.

Finally, the long-term colonization and systemic response to SARS-COV-2 may lead to the impairment of the orienting and executive control networks. Studies have confirmed the persistent neuroinvasive nature of SARS-COV-2(Paniz-Mondolfi et al., 2020), which may cause neurometabolic disorders with long-term changes, such as systemic inflammatory factor and cytokine hyperactivation, destruction of endothelial cells, significant increases in glutamate levels, glial dysfunction, and axonal dysfunction (Leng et al., 2023; Monje & Iwasaki, 2022). However, the exact mechanism underlying this is unknown, and further research is needed.

Our study has some limitations. First, in the current study, although we used the HAMD and HAMA scales to eliminate the effects of anxiety and depressive mood on the cognitive tests, they were not completely avoided. Second, we did not conduct quality of life or cognitive assessments in the patients prior to infection with SARS-COV-2, which prevented us from being able to determine the magnitude of the change in individual patients. However, this is a limitation of any study on an acute, unpredictable medical condition (e.g., COVID-19). In addition, our study was conducted among HCWs with mild COVID-19. It represents only a specific category of the population and cannot be generalized to all non-hospitalized patients with COVID-19. Finally, the sample size of our study was small. Future longitudinal studies with larger sample sizes from multiple centers are needed to further explore the pathophysiological mechanisms by which SARS-COV-2 affect attentional function. Future studies may also explore the mechanisms of the specific neural effects of SARS-COV-2 on other cognitive functions, such as memory, decision-making, executive function, and information processing speed, in conjunction with other indices of rs-fMRI.

## Conclusion

The neural mechanisms underlying impairment of attentional function are intricate and complex. This study focused on three attentional subnetworks and alterations in brain activity using the ANT and rs-fMRI to explore the causes of attentional impairment in HCWs after mild COVID-19 and provide insights for subsequent research on attentional impairment caused by COVID-19. It will also help to improve the care of HCWs mildly infected with SARS-COV-2.

## Data Availability

The data presented in this study are available on request from the corresponding author.
